# Air Quality in Alternative Housing Systems May Have an Impact on Laying Hen Welfare. Part II—Ammonia

**DOI:** 10.3390/ani5030389

**Published:** 2015-09-03

**Authors:** Bruce David, Cecilie Mejdell, Virginie Michel, Vonne Lund, Randi Oppermann Moe

**Affiliations:** 1 Norwegian Veterinary Institute, P.O. Box 750 Sentrum, 0106 Oslo, Norway; E-Mail: cecilie.mejdell@vetinst.no; 2 French Agency for Food, Environmental and Occupational Health Safety (Anses), BP 53, 22440 Ploufragan, France; E-Mail: virginie.MICHEL@anses.fr; 3 Norwegian University of Life Sciences, Pb 8146 Department, 0033 Oslo, Norway; E-Mail: randi.moe@nmbu.no

**Keywords:** furnished cages, loose housing, aviaries, behaviour, health, poultry

## Abstract

The EU ban on conventional barren cages for laying hens from 2012 has improved many aspects of laying hen welfare. The new housing systems allow for the expression of highly-motivated behaviors. However, the systems available for intensive large-scale egg production (e.g., aviaries, floor housing systems, furnished cages) may cause other welfare challenges. We have reviewed the literature regarding the health, behavior, production characteristics, and welfare of laying hens when exposed to ammonia in their housing environment. Concentrations of ammonia gas are commonly high in aviaries and floor housing systems in which manure is not regularly removed, whereas they are usually lower in furnished cages. High levels are found during the cold season when ventilation flow is often reduced. Ammonia is a pungent gas, and behavioral studies indicate chickens are averse to the gas. High concentrations of gaseous ammonia can have adverse health effects and, when very high, even influence production performance. The most profound effects seen are the occurrence of lesions in the respiratory tract and keratoconjunctivitis. There is also evidence that high ammonia concentrations predispose poultry to respiratory disease and secondary infections. We conclude that there are animal welfare challenges related to high ammonia levels, and that immediate actions are needed. Development of improved systems and management routines for manure removal and ventilation will be important for the reduction of ammonia levels and hence will contribute to safeguarding hen welfare.

## 1. Introduction

The EU ban on conventional cages from 2012 (EU council directive 1999/74/EC) has improved the welfare of laying hens. The use of furnished cages allows for the expression of behaviors such as perching, nesting, and dust bathing, whilst loose housing systems additionally allow for activities such as walking, wing flapping, and foraging. The systems available for intensive large-scale egg production (e.g., aviaries, floor housing, and furnished cages) may, however, have side-effects that cause other welfare problems [1]. In two review articles in this special issue on poultry welfare, we discuss the effects of air quality in the housing systems available after the ban on conventional barren cages. Effects on health, behavior, and production performance in laying hens, with emphasis on dust [2] and ammonia (reported here), are reviewed. When relevant information is lacking or scarce for laying hens, references from studies in other poultry species (e.g., broilers and turkeys) are included in the review.

## 2. Ammonia in Poultry Houses

Ammonia is an invisible, water-soluble alkaline gas that is recognized as one of the most prominent contaminants in buildings housing laying hens [3]. It can normally be detected by humans at a concentration of 10 ppm because of its pungent smell and irritating effect on the eyes and the mucous membranes of the upper respiratory system [4]. After prolonged or frequent exposure, humans may develop a chronic sinusitis which desensitizes them to the smell of ammonia, thus causing an inability to detect ammonia before it reaches much higher concentrations [5,6].

## 3. Source

Most ammonia originates from the decomposition of the nitrogen-containing excretion from the kidneys and the gut of the bird [7]. Nitrogen is excreted as uric acid (80%), ammonia (10%), and urea (5%). Once excreted, uric acid and urea are readily converted to ammonia by a series of microbial enzymes present in the manure and by microbial degradation. Water (or moisture) is needed for the growth of microorganisms and functioning of enzymes to convert uric acid to urea. *Bacillus pasteurii* is one of the primary uricolytic bacteria that facilitate ammonia production in the litter [8,9].

## 4. Factors Affecting Ammonia Levels in Poultry Houses

The type of manure management system clearly affects ammonia emission. Concentrations of ammonia are generally higher in all types of housing systems with manure composting inside the house. The accumulation of manure is common practice in loose housing systems with litter, although some modern aviaries remove manure regularly by using belts or scrapers. In bedded-floor housing, slats may collect manure under feeding and resting areas whereas other houses have a pit below where fans dry the manure. Table 1 shows a comparison of results from a number of studies measuring ammonia in different housing systems for laying hens. It can be seen that caged systems, both traditional cages and furnished cages, usually have lower concentrations of ammonia compared to floor systems and aviaries. For comparison, the ranges of ammonia concentration for deep litter broiler houses in the UK were reported to be 20–52 ppm in the winter and 12–25 in the summer [10]. A recent study comparing different housing systems on the same farm reported relatively low ammonia levels also in the alternative systems, although significantly higher levels in aviaries compared to furnished cages (averaging 6.7 ppm and 2.8 ppm, respectively, see Table 1) [11]. Nevertheless, the ammonia concentrations can, on some occasions, be very high. A Swedish study of 18 randomly-selected laying hen flocks in floor systems revealed that most houses exceeded 25 ppm ammonia, and in some cases went up to 80 ppm [12]. In a Norwegian case study with one furnished cage system, one aviary, and one floor system, marked differences were noted in the winter [13]: in the system with furnished cages, an average ammonia level of 4 ppm was registered. Concentrations in the aviary system were much higher, reaching 40 ppm on cold days. In the deep-litter floor system, where manure also was stored inside the house, ammonia levels were on average 85 ppm with peak daily average exceeding 100 ppm [14].

In general, several factors affect the ammonia content of the air in poultry houses. These factors include litter type, bird activity, stocking density, manure handling, frequency of manure removal, ventilation rate, and the pH, humidity, and surface area of the stored manure [6,9,11,12]. Diurnal variations have been reported, and one study showed that ammonia levels rose during the time of day when the hens (housed in an aviary system) were active on the litter floor [11]. This indicates that the birds’ activities on the litter, often having a high manure content, may play a role in ammonia levels in poultry houses. However, access to litter does not necessarily affect ammonia levels, as Madelin and Wathes [15] found no differences in the concentrations of gaseous ammonia between houses where broilers were raised on litter or on netting floors with manure depositing below the netting.

Ambient temperature outside, as well as inside, the poultry houses, may have profound effects on ammonia levels. Zhao *et al.* [11] reported an inverse relationship between outdoor temperature and ammonia concentrations in the hen houses, probably due to indirect effect of the ventilation, which increases with increasing outdoor temperature. The authors suggested that the reduced ventilation rate in cold weather combined with humid air resulting in greater moisture content in the litter, giving better conditions for bacterial decomposition of uric acid to ammonia. Indeed, Scandinavian studies show that the ammonia concentration may increase dramatically in the winter months [12,13]. This is most likely as a result of ventilation being decreased in order to avoid extra heating costs in colder climates, which indicates that increased humidity due to condensation may also play a role [13]. In more moderate temperature zones, such as in the Mediterranean area, the seasonal variation may be less pronounced [16]. Additionally, high indoor temperature in combination with moist litter may lead to increased ammonia levels [7,14]. Miles *et al.* [17] found that ammonia levels at 40.6 °C were up to seven times greater than at 18.3 °C. Anderson *et al.* [18] noted that, when the litter moisture content rose above 25%, ammonia was present at detectable levels if the temperature was above 16 °C.

**Table 1:** A comparison of results from a selection of studies measuring ammonia in different housing systems for laying hens.

Study	System	Manure Handling	Season and/or Duration	Ammonia (ppm)
Reuvekamp and van Niekerk, 1996 [19]	Floor	Litter (no removal)	December–April	15.2
Wathes *et al.*, 1997 [10]	Conventional cage	Deep pit	Summer and winter	13.5
	Aviary	Litter	Summer and winter	12.3
Groot Koerkamp and Bleijenberg, 1998 [7]	Aviary	Litter and removal by manure belt	5 consecutive 3-week periods	5–30
Tauson and Holm, 2001 [20]	Furnished cage	Removal by manure belt	over 3.5 years	1–2
	Floor	Removal by manure belt	over 3.5 years	5–40
Liang *et al.*, 2005 [21]	Conventional cage	Daily removal by manure belt	1 year	2.8–5.4
	Conventional cage	No removal (high rise system)	1 year	35.9–44.8
Nimmermark, 2009 [13]	Aviary	Litter and weekly removal by belt	January–April	32–38
	Furnished cage	Removal every 5 days by belt	January–April	2.5–5.2
Hinz *et al.*, 2010 [22]	Furnished cage	Weekly removal by belt	Two years	0.4–4.2
	Aviary	Litter and weekly removal by belt	Two years	2.2–18.5
	Aviary	Litter (no removal)	Two years	9.2–47.4
	Floor	Litter (no removal)	Two years	1.9–33.6
Costa *et al.*, 2012 [23]	Conventional cage	Open manure storage	One year	5.37
	Conventional cage	Removal by belt	One year	4.95
	Aviary	Litter and removal by belt	One year	3.85
Zhao *et al.*, 2015 [11]	Conventional Cage	Twice weekly by belt	27 months	4.0
	Furnished cage	Twice weekly by belt	27 months	2.8
	Aviary	Litter and twice weekly by belt	27 months	6.7

## 5. Consequences for Birds

### 5.1. Health

The water solubility of ammonia allows for it to be absorbed in dust particles and litter as well as in mucous membranes [24,25]. This is evidenced by the harmful effects of high concentrations of ammonia on various mucous membranes and on skin in contact with manure. In general, it can be stated that ammonia is toxic to animal cells [24]. Ammonia concentrations above 25 ppm may have adverse effects on the health and production of poultry, the main clinical symptom being keratoconjunctivitis (*i.e.*, inflammation of the cornea and conjunctiva of the eye) [26,27,28,29]. Marthedal [28] reports that young animals are more sensitive to ammonia than mature animals. However, the most profound effects of high concentrations of gaseous ammonia are seen in the respiratory system. Inhalation of gaseous ammonia, aerosols containing NH_3_ dissolved in water, or ammonia bound to dust particles allow for close contact with the epithelia of the respiratory system, thereby causing lesions. Dalhamn [30] proposed that ammonia could impair the mucous flow and ciliary action in the trachea of rats. This has also been demonstrated in poultry, and experiments have shown ammonia to have significant effects on the respiratory tract at concentrations of 25 ppm. These effects include the loss of tracheal cilia and histopathological changes to the tracheal epithelium in chickens [31] and turkeys [32,33,34]. Oyetunde *et al.* [35] demonstrated that very high levels of ammonia (100 ppm) can cause deciliation of the epithelium of the upper portion of the trachea in broiler chickens. However, such high levels are not often seen in egg production facilities. The loss of tracheal cilia results in a decrease in the effectiveness of the mechanical defense mechanism of the respiratory system. The same investigators found that this damage is further perpetuated by an increased mucous secretion. In the same experimental study in which broiler chickens were exposed to 100 ppm ammonia, macroscopic and microscopic lesions in air sacs were observed as early as 12 h post-exposure [35].

The resultant damage to the bird’s ability to withstand infections has been verified by a number of researchers. Anderson *et al.* [18] demonstrated that poultry exposed to 20 ppm ammonia exhibited an increased susceptibility to Newcastle disease. Higher concentrations of ammonia (60–70 ppm) seem to predispose broiler chickens to respiratory disease and secondary infections [36], whilst animals exposed to lower concentrations can be expected to experience more severe damage to the respiratory tract upon challenge by infectious agents [37]. Turkeys exposed to 10 to 40 ppm ammonia and then challenged with *E. coli* had greater amounts of the bacteria in their lungs compared to animals that were not exposed [34]. Even the reaction to live vaccines against infectious bronchitis (IB) and Newcastle disease (ND), seems to be more severe—resulting in the atrophy of the bursa Fabricius [36,37]. Exposure to ammonia may also increase the severity of coccidiosis, whilst long time exposure can result in changes to the spleen, kidneys and adrenal glands [38].

### 5.2. Behavior

It has been demonstrated that, when given the choice of selecting compartments with 0, 10, 20, 30, and 40 ppm ammonia, laying hens chose the fresh air [39]. Broiler chickens, when given a free choice between 4, 11, 20, and 37 ppm atmospheric ammonia over a period of 16 days, avoided the two higher concentrations [40]. These results indicate that the birds experience ammonia as aversive. Both layers and broilers, when given the opportunity, will avoid ammonia in concentrations that are commonly found in poultry houses. Bullis *et al.* [29] reported that in broiler chickens where ammonia was suspected to have caused keratoconjunctivitis, birds were observed huddling in groups, rubbing their eyes against their wings and keeping their eyes closed and showing signs of hypersensitivity to light.

### 5.3. Production

Egg quality may be adversely affected by high ammonia levels as measured by reduced albumen height, elevated albumen pH, and albumen condensation [41]. Another stated effect of high concentrations (100 ppm) is that hens come later into lay and produce fewer but larger eggs [42]. This is believed to be due to a decrease in the rate and depth of respiration causing a change in the blood pH as the effect was also dependent upon temperature—the higher the temperature the shorter the time of exposure necessary to see effects (7–10 weeks at 2–18 °C, respectively). The same authors demonstrated that the effects of ammonia exposure on pullets early in their life may have a lasting effect on them as laying hens. An exposure to 78 ppm ammonia during the growing period caused an increase in the time it took for the hens to reach 50% production and also a reduction in the number of egg-producing days. Exposure to ammonia levels of 200 ppm for 17 days was demonstrated to cause a significant decrease in egg production with the hens exhibiting a reduced feed intake and a loss of weight [43,44]. Furthermore, ammonia at levels of 50 ppm over seven to eight weeks resulted in decreased appetite which, in turn, resulted in a reduced efficiency of food utilization and subsequently lower body weights in broilers [45,46].

## 6. Discussion

Many aspects of laying hen behavior, health, production, and welfare in aviaries, floor housing systems and furnished cages are extensively reviewed by EFSA [1]. The alternative systems are clearly superior to barren battery cages regarding the hens’ freedom of movement, including their fulfillment of several strongly motivated species specific behaviors like nesting, perching, dust bathing, and scratching. This is especially true for loose housing systems. Also, the lower stocking density in loose housing compared to cage systems may result in cooler temperatures in summer, which would have a positive impact on hen welfare. Some of the systems give outdoor access to birds (e.g., organic and free range). This allows hens to perform natural behaviors in fresh air and also has a positive effect on indoor air quality by reducing indoor stocking density. Although EFSA did address challenges with the aerial environment, in general, less attention has been paid to potential welfare problems related to the air quality in the housing systems that are commercially available after the ban of barren cages. In two articles we have reviewed the available literature on potential welfare challenges related to air quality in laying hens with respect to dust [2] and ammonia (reported here).

This review has documented that high ammonia levels sometimes are found in alternative housing systems for laying hens. Although other types of poultry and livestock housing often have more pronounced problems with ammonia levels, we hope that the animal welfare improvements with regards to behavioral and spatial factors do not make us complacent with regards to factors affecting animal health. Fresh air is preferred by birds, and they show behavioral responses indicative of discomfort, which clearly indicates that they experience a high level of atmospheric ammonia as aversive. Furthermore, detriments to health further stress the negative effects of high concentrations of ammonia on laying hen welfare. The duration of exposure to unpleasant experiences is of importance to welfare. It is therefore necessary to consider that laying hens have a longer life span than e.g., broilers and turkey, further emphasizing the importance of keeping ammonia levels at a low concentration throughout the laying hens’ life.

A limited number of studies indicate that furnished cages may be more favorable regarding air quality than aviaries and floor housing systems. Since the source of ammonia is the manure, it is obvious that novel systems for litter removal and improved management routines need to be developed and implemented. In hen houses where high concentrations of ammonia are found, the manure is commonly stored inside the building. There is no doubt that ammonia concentrations would be lower if most of the bird droppings could be kept away from the litter area and removed from the building on a daily basis, and such systems should be prioritized in new technological developments. A transition to hen house systems with manure belts underneath the enriched cages and floorages in aviaries, with daily manure removal will improve air quality [47,48,49] and is welcomed. Additionally, for the litter area, systems enabling replacement of litter on a regular basis should be implemented.

A reduced ventilation rate during the cold season, to maintain a suitable indoor temperature, combined with manure being stored inside the hen house, is the main cause of ammonia sometimes reaching very high levels. The development of energy-efficient heating systems allowing for appropriate ventilation rates bringing down ammonia levels even during the winter season should, therefore, be encouraged. Heating systems and heating costs, respectively, should be accepted by the farmer to be standard investment and running expenses. The drying rate of the manure is important to reduce ammonia emission, since enzymatic and microbial degradation of uric acid/urea to ammonia are dependent on moisture. Minimum emission from manure can be achieved if a dry matter content of 60% is reached within 50 h after excretion of the feces [50]. Poorly-managed drinkers cause water loss and spillage and represent challenges for keeping the litter dry. Furthermore, more knowledge regarding absorbent litter materials and litter management procedures that help keep the manure dry would also help to reduce the production of ammonia. There are also additives to spray on or mix with litter which may act to reduce ammonia emissions [48,51,52]. Even dietary manipulations may reduce ammonia emissions from manure [52].

The fact that there is a large variation of ammonia levels reported from alternative commercial hen houses shows the high potential for improvement. Systematic studies on the success factors are welcomed, and an integrated approach with close cooperation between experts in technology and biology (e.g., veterinarians, feed experts and ethologists) would help to find optimal solutions.

The findings of Oyetunde *et al.* [35] show that there may be a substantial synergism between various components that reduce air quality, and a clear synergy between ammonia and dust, impairing pulmonary function, has been reported in poultry workers [53]. However, studies of the synergistic effects of dust and ammonia on laying hen health are, in general, lacking.

It should also be noted that levels of aerial contaminants observed in poultry and other livestock facilities are often several times higher than the accepted levels for human exposure at the workplace. Exposure concentrations for humans associated with significant pulmonary function decrements were reported to be 12 ppm for ammonia [54]. According to European regulations (COMMISSION DIRECTIVE 2000/39/EC, Council Directive 98/24/EC) occupational exposure limit values are set to 20 ppm and 14 mg/m^3^ of ammonia for 8 h exposure. In France, the limit is 10 ppm or 7 mg/m^3^ of ammonia. The occupational exposure limit (OEL) value for 8 h work in Norway is 25 ppm. These exposure limits were exceeded in the multilevel and floor housing systems, although the concentrations were occasionally below the OEL in the multilevel system [13]. Thus, measures ensuring a better environment for the laying hens will therefore also be beneficial for their caretaker. In situations where the air quality is particularly poor, it becomes less pleasant for the caretakers to be among the birds. They spend less time in the henhouse which, in turn, makes it less likely that any health and welfare problems in the flock are detected at an early stage. Furthermore, birds may become more fearful of humans due to less contact, affecting both welfare and production negatively [55,56].

## 7. Conclusions

The two literature reviews on dust and ammonia, respectively, indicate that alternative housing systems for laying hens may pose a challenge with regard to dust and ammonia concentrations. The increased activity of the hens successfully achieved in these systems, is unfortunately exaggerating the problem. In order to safeguard the intended welfare benefits of the recent ban on barren cages, both immediate and long-term actions are needed to contribute to the reduction of air pollutants by other measures. Important solutions to reduce ammonia levels involve development and implementation of optimal systems for manure removal and for ventilation, which may be of particular importance under cold climatic conditions in e.g., the Nordic countries. Close collaboration between experts within fields of technology and biology should be encouraged to develop good technical solutions and management procedures that reduce ammonia concentrations and other pollutants.
